# Free-Weight Resistance Exercise Is More Effective in Enhancing Inhibitory Control than Machine-Based Training: A Randomized, Controlled Trial

**DOI:** 10.3390/brainsci10100702

**Published:** 2020-10-03

**Authors:** Jan Wilke, Vanessa Stricker, Susanne Usedly

**Affiliations:** Department of Sports Medicine, Goethe University, Ginnheimer Landstraße 39, 60487 Frankfurt am Main, Germany; vanessastricker@aol.com (V.S.); s.usedly@t-online.de (S.U.)

**Keywords:** resistance training, cognition, barbell training, strength training

## Abstract

Resistance exercise has been demonstrated to improve brain function. However, the optimal workout characteristics are a matter of debate. This randomized, controlled trial aimed to elucidate differences between free-weight (RE_free_) and machine-based (RE_mach_) training with regard to their ability to acutely enhance cognitive performance (CP). A total of *n* = 46 healthy individuals (27 ± 4 years, 26 men) performed a 45-min bout of RE_free_ (military press, barbell squat, bench press) or RE_mach_ (shoulder press, leg press, chest press). Pre- and post-intervention, CP was examined using the Stroop test, Trail Making Test and Digit Span test. Mann–Whitney U tests did not reveal between-group differences for performance in the Digit Span test, Trail Making test and the color and word conditions of the Stroop test (*p* > 0.05). However, RE_free_ was superior to RE_mach_ in the Stroop color-word condition (+6.3%, *p* = 0.02, R = 0.35). Additionally, RE_free_ elicited pre-post changes in all parameters except for the Digit Span test and the word condition of the Stroop test while RE_mach_ only improved cognitive performance in part A of the Trail Making test. Using free weights seems to be the more effective RE method to acutely improve cognitive function (i.e., inhibitory control). The mechanisms of this finding merit further investigation.

## 1. Introduction

For millennia, resistance exercise (RE) has represented an essential type of physical training, evoking manifold benefits in a plethora of populations and conditions [1,2,3,4,5]. For instance, RE has been shown to counteract age-related sarcopenia [1], reduce arterial blood pressure [2], improve recovery from musculoskeletal disorders [3] and increase sports-related motor performance [4,5]. Most studies examining the effects of RE predominantly focused on peripheral adaptations in the soft tissue, i.e., the cross-sectional area of the skeletal muscles. However, beyond this, RE also appears to impact brain function. Pooling the results from 12 trials, a recent multilevel meta-analysis concluded that a single training bout acutely increases specific sub-domains such as inhibitory control and cognitive flexibility [6].

Despite the established knowledge about the general interaction between RE and brain function, the moderators driving the changes in cognitive performance (CP) are a matter of debate. Studies addressing this issue have yielded heterogeneous results and a high level of uncertainty regarding the influence of modifiable (e.g., intensity, duration) and non-modifiable (e.g., age) factors [6]. Besides these quantitative variables, Pesce [7] suggested to focus on qualitative characteristics. In fact, studies using electroencephalography or near-infrared spectroscopy have revealed that cortical activations patterns and hemodynamics vary as a function of task complexity (e.g., stable repetitive monotonous vs. variable or alternating, coordinatively challenging activities; [8,9,10,11]). The question arises if changes such as higher perfusion and/or a facilitation of specific sensorimotor cortices could be beneficial for cognitive performance.

Some researchers investigated the impact of varying task complexity on brain function. In adolescents attending an elite performance school, Budde et al. [12] found a 10-min, coordinatively challenging exercise session (e.g., bouncing one or two balls, alternatingly and/or simultaneously, concurrently passing balls with foot and hand) to acutely enhance executive function to a greater degree than a traditional intervention of identical duration (general moderate-intensity exercise without major coordinative demands). Gallotta et al. [13] made opposite findings. They instructed primary school children (8–11 years) to either participate in a normal school lesson lacking physical activity, a regular non-enriched physical education session (e.g., walking, running, skipping) or a coordinatively enriched physical education session (e.g., basketball mini-games with varying rules fostering decision-making). Interestingly, the coordinatively challenging session was least effective.

Hitherto, no study has examined the cognitive effects of task complexity in RE. However, in a pioneering trial, Carraro et al. [14] found free-weights training to more strongly increase arousal (a psychological state of being attentive) than machine-based training. As exercise-induced gains in arousal are linked to better CP [15], the objective of the present study was to test the hypothesis that free-weight RE would enhance cognition more effectively than a quantitatively matched intervention with machines.

## 2. Materials and Methods

### 2.1. Ethical Standards and Study Design

The study is nested within the COINS (COgnition and INjury in Sports) network project. A two-armed, randomized, controlled trial was performed. It was prospectively registered at the German Register of Clinical Trials (DRKS00022281) and conducted in accordance with the Declaration of Helsinki including its recent modification of Fortaleza (2013). Ethical approval (ref. 2020-39) was obtained on 7 June 2020 from the ethics committee of the Faculty of Psychology and Sports Sciences of Goethe University Frankfurt, and each volunteer provided written informed consent.

Participants were randomly allocated to two groups: (1) resistance training with free weights (RE_free_) or (2) resistance training with machines (RE_mach_). Prior to and after the intervention, cognitive performance was assessed. All participants visited the laboratory twice with 5 to 7 days in-between. While the first session served for familiarization with the tests and the training equipment, the actual intervention was performed on the second appointment. Randomization was performed using the software package “BiAS for Windows”, version 9.05 (Goethe University, Frankfurt, Germany).

### 2.2. Participants

Healthy adults (*n* = 46, 27 ± 4 years, 26 men) were recruited between June and August 2020 by means of personal contact and poster advertising at the local university campus. They had to engage in a minimum of five sporting hours per week. Exclusion criteria were (a) severe orthopedic, cardiovascular, pulmonary, neurological, psychiatric or inflammatory rheumatic diseases; (b) pregnancy or nursing period; (c) analgesic intake during the trial or in the 48 h prior to study enrollment; (d) impairments in color vision and (e) history of surgery or trauma in the lower extremity.

### 2.3. Intervention

The interventions and related procedures have been validated in a previous trial assessing the impact of RE on arousal [14]. When allocated to RE_mach_, the participants completed a resistance training session using conventional training machines (shoulder press, leg press and chest press). Exercises for individuals in RE_free_ (military press, back squat, bench press) were executed with a barbell. Repetition numbers, set durations and relative intensities were identical in both groups. Participants performed four sets with the descending pyramid system (six, eight, ten, and twelve repetitions, weight progressively decreasing from set to set). To standardize movement velocity, the concentric phases lasted one second and the eccentric phases two seconds. Rest duration between-sets was 115 s. Weights were determined according to the individual 6-repetition maximums (6RM) of the respective exercises, which was determined during the familiarization session [14]. Prior to testing, participants performed two warm-up sets at 25% and 50% of the anticipated 1RM [16]. These sets, together with previous training records, were used to determine the starting weight. In the actual measurements, weight was increased once six correct repetitions had been performed. The interval between sets was 180 s [17]. Only assessments with a maximum of 5 attempts were considered valid [18]. In our sample, most participants required 1–3 sets (range 1–4 sets). All workouts were monitored by investigators holding an academic degree in Sports Sciences. Verbal feedback was continuously given if errors in movement execution were observed.

### 2.4. Outcomes

Immediately before and after the intervention, markers of CP were measured. To prevent practice effects, three strategies were used [19]. Firstly, in the familiarization session, all participants completed three repetitions of each test. Secondly, prior to initiating the actual assessments, one warm-up trial was performed. Finally, no identical tests forms (different color/number orders) were applied. Testing order was randomized and the delay between the end of the experimental condition and the start of the post-measurements was standardized amounting to 60 s.

Assessments included three tests. The *Stroop task* has three parts. In the first and second which both capture attention, the participants were required to name words written (S_w_) or colors (S_c_) displayed on a sheet as quickly as possible. The third section, a measure of inhibition control (S_cw_), consisted of color words presented incongruently (e.g., “green” written in red or “blue written in yellow). Here, the participants needed to name the color of the word while ignoring the letters. In all three parts, time until task completion was documented. The Stroop test exhibits high reliability (ICC: 0.82) and internal consistency (Cronbach’s alpha: 0.93 to 0.97) [20].

The *Trail Making test* (TMT) has two parts. In part A, the participants were required to connect successive numbers using a pen at maximal possible speed (e.g., 1–4). In part B, numbers and letters (e.g., 1 to a to 2 to b) were to be linked in an alternating manner. Similar to the Stroop test, time needed for completion was recorded. The results are suggested to represent a measure of visual screening/attention (TMT-A) and cognitive flexibility/working memory (TMT-B). High reliability (ICC: 0.81 to 0.86) and construct validity of the TMT have been demonstrated [21,22].

In the *Digit Span* (DS) test, two conditions were performed. In the first, the participants had to recall and repeat increasing amounts of numbers read to them. Initially, four numbers were to be memorized. In case of success, five numbers were named. For each step, two repetitions were performed and one or zero points awarded depending on recall success. The test ends if both trials were failed. The second condition was identical to the first, but the numbers had to be repeated in reversed order (e.g., 2,4,7,9 becomes 9,7,4,2). Both test parts and the composite score were linked to short-term and working memory [23]. The DS test is reliable for repeated measurements (*r* = 0.73; [24]).

Prior to starting outcome assessments, subjective arousal (Likert scale from “0—not activated” to “6—highly activated”), concentration (10 cm Visual Analogue Scale, 0 = not concentrated at all to 10 = highly concentrated) and heart rate (heart rate monitor) were assessed. Additionally, after the interventions, the participants stated their rate of perceived exertion (6–20 RPE scale [25]) as well as enjoyment of the intervention (Likert scale from “0—not fun at all” to “6—most possible fun”).

### 2.5. Data Processing and Statistics

Kolmogorov–Smirnov analyses revealed violations of the normalcy assumption. To identify relative pre-post changes of CP within groups, we constructed parameter-free 95% confidence intervals [26] while between-group differences were detected by means of the Mann–Whitney U test. With regard to the latter, in case of significance, effect sizes (R = Z/sqr(n)) were computed according to Rosenthal [27] and interpreted as small (R = 0.1), moderate (R = 0.3), large (R = 0.5) or very large (R > 0.7). To reveal potential moderators of the intervention effect (age, sex, BMI, physical activity volume, arousal, exercise enjoyment, subjective exertion during exercise), we used Kendall’s tau correlation. *p*-values < 0.05 were considered to be significant in all calculations, the software used was “BiAS for Windows”, version 9.05 (Goethe University, Frankfurt, Germany).

## 3. Results

Both groups showed comparable cognitive performance at baseline and were not different regarding age, sex, BMI, physical activity and pre-exercise arousal (*p* > 0.05, Table 1). All participants completed the disposed interventions without the occurrence of adverse effects.

### 3.1. Exercise Effects on Cognitive Performance

**Figure 1:**
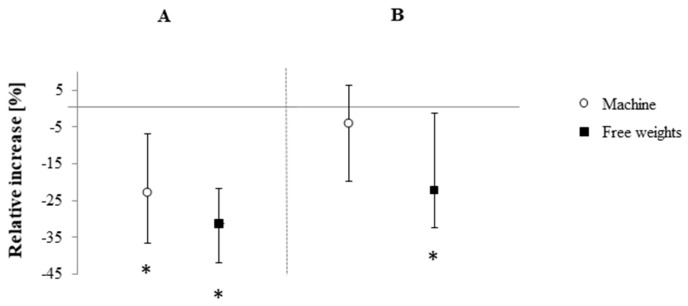
Relative changes in the two parts (**A**,**B**) of the Trail Making test (TMT) following free-weight and machine-based resistance exercise. Displayed are medians and parameter-free 95% confidence intervals. * = difference to pre-intervention value according to 95% confidence intervals

**Figure 2:**
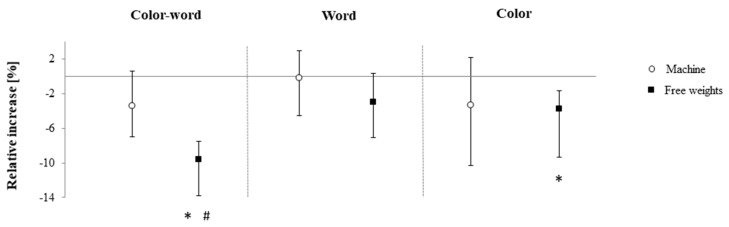
Relative changes in the three conditions of the Stroop test following free-weight and machine-based resistance exercise. Displayed are medians and parameter-free 95% confidence intervals. * = difference to pre-intervention value according to 95% confidence intervals, # = significant difference of pre-post change when compared to the other group.

No differences between groups were found for DS, TMT as well as S_c_ and S_w_ (*p* < 0.05, Table 2). However, analysis of the confidence intervals revealed that RE_free_ increased TMT-A (+34.6%), TMT-B (+24.3%), S_w_ (+2.9%) and S_c_ (+4.2%) performance relative to baseline, while RE_mach_ only improved TMT-A (+23.5%, Figure 1 and Figure 2). Additionally, RE_free_ improved S_cw_ both pre to post according to the confidence intervals (+9.6%) and in comparison to RE_mach_ (+6.3%, *p* = 0.02, R = 0.35; moderate effect size, Figure 2).

### 3.2. Potential Moderators

Exhaustion, enjoyment as well as changes in arousal and heart rate were not different between groups (*p* < 0.05). Additionally, none of these variables correlated with changes in cognitive performance (*p* < 0.05).

## 4. Discussion

A wealth of evidence supports the beneficial short-term impact of resistance exercise on cognitive performance [6]. However, so far, research on the moderators of this effect predominantly focused on training intensity [28,29,30,31,32] while the relevance of exercise characteristics, i.e., the type of RE, had been scarcely examined. In detail, available studies mostly investigated machined-based (e.g., [33,34,35,36]) or a combination of machined-based and free-weight RE (e.g., [37,38,39,40,41,42]) but none provided a direct comparison of different regimes. The present trial reveals that the magnitude of exercise-induced CP improvements following free-weight RE is substantially larger when compared to machine-based training.

Several mechanisms potentially mediating short-term CP changes following RE have been discussed. A previous study demonstrated free-weight exercise to induce higher arousal levels when compared to machine-based training [14]. However, this finding could not be replicated in our trial as arousal was not different between groups. A potential reason for this could be that we used a 10-point Likert scale, while Carraro and colleagues had applied the Felt Arousal Scale with six points. Irrespectively, the impact of arousal as an effect trigger in different RE types requires further examination. Cerebral blood flow represents another factor suspected to explain activity-induced CP improvements. Experiments using aerobic exercise showed an intensity-dependent increase of brain perfusion until the ventilatory threshold [43]. Only few studies examined this association for RE, but initial evidence points towards the existence of blood flow fluctuations during exercise which may facilitate CP [43,44]. Together with or in absence of perfusion changes, altered cortical activation patterns may be triggered by RE. In both, younger adults and individuals with mild cognitive impairment, increased P3 amplitudes (linked to activity and cognitive functioning) were detected immediately following an exercise bout [45,46]. Finally, exercise generally modulates the production of cortisol and moderately elevated concentrations of the stress hormone can enhance working memory [47]. Evidence, however, is ambiguous for RE and both acute decreases [38] and increases [48] have been reported. While all these findings are intriguing, none of the mechanistic studies particularly addressed the question as to whether the magnitude of the metabolic, circulatory and electrophysiological changes is dependent on the RE characteristics (i.e., RE with free weights vs. RE with machines). Based on the available evidence showing an association of task complexity and cortical activation [8,9,10] and our data, we speculate that training with free weights may require higher levels of concentration, sensory processing and motor coordination and with this, higher or more complex brain activation levels than similar interventions using training machines.

RE with free-weights, in contrast to machine-based RE, improved S_c_ and TMT-B performance but no significant between-group differences were found. Some uncertainty therefore remains regarding a higher effectiveness of free-weight exercise in enhancing simple cognitive functions such as attention, processing speed, reaction time and visual scanning. Contrarily, a moderate effect indicating superiority of RE with free weights was found for inhibitory control (S_cw_). This finding seems plausible as exercising with a barbell requires constant fine and gross motor adaptations and corrections of the ongoing movement. Improvements of inhibitory control may be of relevance in a variety of contexts. Previous studies found that poor performance during response inhibition tasks is associated with the risk of future falls in community-dwelling older adults and individuals with neurological diseases [49,50,51,52]. Health professionals may hence consider incorporating free-weight exercises when designing exercise programs for seniors or patients. Beyond this, using free- or bodyweight resistance exercise could also be of value as a warm-up for athletes from both an injury-preventive and a performance-related perspective. Giesche et al. [53] examined the relation between inhibitory control and dynamic postural control during unplanned single-leg landings in healthy active individuals. Participants with low S_cw_ values had higher center of pressure path lengths, which is indicative of a possible stability deficit. With regard to sporting success, several studies [54,55] have shown an association between inhibitory control and game performance sports.

Although machine-based paradigms are frequently used in studies examining the effects of RE on CP, R_mach_ did only improve TMT-A performance but, as said, had no effects on measures of executive function (TMT-B, S_cw_) or other tests capturing lower order cognitive skills. Compared to S_c_ and S_w_, the TMT-A places higher demands to visual search [56], and hence, it may be speculated that RE_mach_ rather improves this skill than processing speed and reaction time in general.

Our results call for further research. (1) From a general point of view, gaining insight into the mechanisms explaining the higher effectiveness of R_free_ in increasing CP should be a paramount objective of future trials. (2) In detail, we found a superiority of R_free_ in inhibitory control, which is typically classified as a higher-order cognitive function. As the analysis of the confidence intervals also revealed pre-post improvements in lower-order functions (e.g., processing speed and visual scanning), which did not occur following machine-based training but failed statistical significance in the group comparison, we recommend follow-up experiments focusing on this area. Finally, it would be of interest to compare the traditional RE methods studied here with newer approaches such as high-intensity functional training, which mixes characteristics of endurance and resistance exercise.

Some limitations need to be discussed. Analysis of our data showed that it was non-normally distributed. As the choice of the sample size had been based on a biometric calculation for parametric testing and as non-parametric testing is more conservative, our trial may have had a slight lack of power preventing the detection of small-magnitude differences. When considering the confidence intervals, this, e.g., may relate to performance in the Trail Making test. Two other issues relate to the comparability of the interventions. Firstly, although we matched training intensity relative to individual maximal strength, exercising with free weights is known to achieve higher muscle activations [57]. Secondly, we did not use a linear encoder to control movement velocity. Minor differences may hence have occurred between the training regimes.

## 5. Conclusions

Resistance exercise performed with free weights is more effective in acutely increasing inhibitory control than the use of conventional training machines. It may hence be of interest for both, elderly individuals aiming to prevent falls or athletes seeking to improve performance.

## Figures and Tables

**Table 1:** Characteristics of the two groups measured pre- and post-intervention.

	RE_mach_		RE_free_		Total Pre	Total Post
	*Pre*	*Post*	*Pre*	*Post*		
Age (yrs.)	27.0 ± 4.3		27.3 ± 4.4		27.2 ± 4.3	
Sex	12♂, 11♀		14♂, 9♀		26♂, 20♀	
Physical Activity (hrs./week)	8.2 ± 3.8		7.8 ± 3.6		8.0 ± 3.7	
BMI	22.7 ± 2.5		23.8 ± 3.1		23.3 ± 2.8	
Arousal (0–10)	6.2 ± 1.6	7.1 ± 1.4	6.5 ± 1.5	7.0 ± 1.7	6.3 ± 1.5	7.1 ± 1.6
Heart rate (bpm)	79.2 ± 15.5	103.4 ± 17.6	77.3 ± 9.7	104.9 ± 18.4	78.2 ± 12.8	104.1 ± 17.8
Enjoyment (0–10)		6.8 ± 2.1		7.6 ± 2.0		7.2 ± 2.0
Subjective exertion (6–20)		15.2 ± 2.1		15.1 ± 2.2		15.2 ± 2.1

Table shows means and standard deviations. RE_mach_ = machine-based resistance training, RE_free_ = free-weights resistance training, yrs= years, hrs= hours, bpm = beats per minute.

**Table 2:** Cognitive performance measured pre- and post-intervention in both groups.

	RE_mach_		RE_free_	
	*Pre*	*Post*	*Pre*	*Post*
Stroop word (s)	25.9 (24.6–28.1)	25.9 (24.2–28.5)	27.2 (24.0–30.6)	25.7 (24.4–29.7)
Stroop color (s)	31.2 (28.9–35.5)	29.3 (26.8–33.1)	33.1 (28.3–35.7)	30.2 (27.5–34.9) *
Stroop color-word (s)	45.9 (42.2–52.3)	44.7 (40.9–48.1)	49.7 (42.6–56.1)	43.9 (38.3–49.0) *#
TMT-A (s)	25.5 (21.2–34.3)	19.5 (16.3–25.2) *	28.3 (24.7–32.6)	18.5 (16.6–23.2) *
TMT-B (s)	34.3 (28.7–39.1)	30.3 (23.5–38.6)	33.7 (27.4–41.7)	25.5 (21.7–38.1) *
Digit Span (pts.)	11 (10–13)	11 (8–12)	13 (8–14)	12 (10–14)

Table shows medians and (interquartile ranges). RE_mach_ = machine-based resistance training, RE_free_ = free-weights resistance training, s = seconds, pts = points, * = difference to pre-intervention value according to 95% confidence intervals, # = significant difference of pre-post change when compared to the other group.
